# Computerized tongue image segmentation *via* the double geo-vector flow

**DOI:** 10.1186/1749-8546-9-7

**Published:** 2014-02-08

**Authors:** Miao-Jing Shi, Guo-Zheng Li, Fu-Feng Li, Chao Xu

**Affiliations:** 1 The Key Laboratory of Machine Perception (MOE), Peking University, Beijing, P.R., China; 2 Department of Control Science & Engineering, Tongji University, Shanghai, P.R., China; 3 Laboratory of Traditional Medical Syndromes, Shanghai University of Traditional Chinese Medicine, Shanghai, P.R., China

## Abstract

**Background:**

Visual inspection for tongue analysis is a diagnostic method in traditional Chinese medicine (TCM). Owing to the variations in tongue features, such as color, texture, coating, and shape, it is difficult to precisely extract the tongue region in images. This study aims to quantitatively evaluate tongue diagnosis *via* automatic tongue segmentation.

**Methods:**

Experiments were conducted using a clinical image dataset provided by the Laboratory of Traditional Medical Syndromes, Shanghai University of TCM. First, a clinical tongue image was refined by a saliency window. Second, we initialized the tongue area as the upper binary part and lower level set matrix. Third, a double geo-vector flow (DGF) was proposed to detect the tongue edge and segment the tongue region in the image, such that the geodesic flow was evaluated in the lower part, and the geo-gradient vector flow was evaluated in the upper part.

**Results:**

The performance of the DGF was evaluated using 100 images. The DGF exhibited better results compared with other representative studies, with its true-positive volume fraction reaching 98.5%, its false-positive volume fraction being 1.51%, and its false-negative volume fraction being 1.42%. The errors between the proposed automatic segmentation results and manual contours were 0.29 and 1.43% in terms of the standard boundary error metrics of Hausdorff distance and mean distance, respectively.

**Conclusions:**

By analyzing the time complexity of the DGF and evaluating its performance via standard boundary and area error metrics, we have shown both efficiency and effectiveness of the DGF for automatic tongue image segmentation.

## Background

Simple, non-invasive, and inexpensive visual inspection of the human tongue has been a unique diagnostic method of traditional Chinese medicine (TCM) [1], through observing any abnormal changes in the tongue properties and coating. Clinical studies have suggested relationships between visceral cancers, heart diseases, and abnormalities of the tongue and its coating [2-4]. However, the current practice in TCM is mainly subjective, and the quality of the visual inspection varies among medical professionals. Thus, it is beneficial to devise objective quantitative evaluation methods for the color, texture, and surface of the tongue and define their relationships with patients’ health conditions [5,6].

Recently, the development of an automated digital tongue diagnostic system was attempted. Chiu [7] built a computerized tongue examination system, in which the colors of the tongue and the thickness of its coating were identified by a proposed chromatic algorithm. Cai [8] and Li and Cai [9] constructed a digital imaging system to capture tongue images and extract various features for tongue analysis. Zhang et al. [10] demonstrated a novel computer-aided tongue diagnosis system, in which a standard data acquisition device and a new color correction method were used to capture tongue images. Some other computerized systems, such as the tongue computing model, computerized system for tongue diagnosis, and automatic tongue diagnosis system, were also built in [11-13], of which Gao et al. [12] established a mapping relationship between quantitative tongue features and diseases via the support vector machine and obtained promising performances.

**Figure 1:**
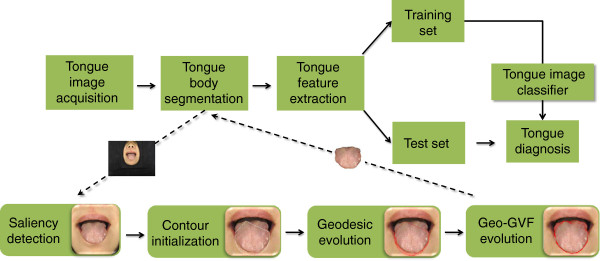
Outline of a computerized model for tongue diagnosis and a flowchart of the proposed DGF for tongue image segmentation.

A general computerized tongue diagnostic system is shown in Figure 1. This system mainly executes four tasks: tongue image acquisition; tongue image segmentation; tongue feature extraction; and tongue image classification. Figure 1 shows the tongue body segmentation as a bridge that combines the acquisition of the tongue image with the feature extraction. Tongue features, such as color, texture, coating, and shape, create difficulties for accurate segmentation of tongue images. Recent studies have mainly focused on the active contour model (ACM), or “Snake”, which evolves a curve by minimizing a specifically defined energy function [14]. The curve is initialized manually or automatically, and propagated to the real boundary on both the internal and external forces [15].

Studies using the ACM have included the general gradient vector flow [16], double snake [17], level set [18], and C^2^G^2^FSnake [19]. Owing to the special characteristics of the tongue color, different color channels have been embedded into these segmentation methods, such as region growing [20], fuzzy C-means [21], and shortest path [22].

**Figure 2:**
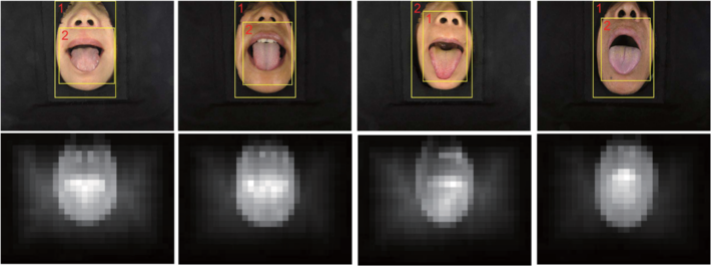
**Saliency detection in tongue images.** Top row: images and saliency objects detected by the saliency object detector; bottom row: corresponding saliency maps of the four images.

In our previous work [19], C^2^G^2^FSnake was proposed as a novel approach for automatic tongue segmentation, using prior location of the tongue body for active contour initialization. It embedded the color space information into the active contour evolution, and the segmentation precision was thus enhanced. Nevertheless, there remained difficulties associated with background removal in clinical images (Figure 2) and parameter selection.

Regarding the background removal, a saliency object detector [23] was adopted to refine the clinical tongue image. This was an unsupervised method that was easier to implement compared with the method of Fu et al. [15]. For the parameter selection, a novel computerized tongue image segmentation method named the double geo-vector flow (DGF) was proposed based on the unique shape of the tongue body and its location on the face. The saliency window was refined and the active contour was initialized for the upper and under parts accordingly. The geodesic flow first propagated the under part, the geo-gradient vector flow (Geo-GVF) then took over the work, and the upper curve was evolved to the real boundary. Based on its ability to split, the geodesic flow showed superior flexibility compared with the parameterized model used by Shi et al. [19], and the geometric term in the gradient vector flow (GVF) speeded up the curve propagation for the upper part and maintained its accuracy.

The tongue images were captured in a closed darkroom that shielded against light disturbance from the outside, and the position of the camera was flexible and adjustable, thus avoiding color distortion caused by light interference. A clinical tongue image database has already been collected by Shanghai University of TCM.

This study aims to devise an objective approach and quantitative model to evaluate automatic tongue segmentation in a computerized tongue diagnostic system.

## Methods

In this study, an active contour-based tongue image segmentation method was proposed. First, the tongue image was refined by a saliency window, and the active contour was initialized by taking advantage of the prior knowledge of the tongue image. Second, the tongue area was initialized into two parts: the binary upper part and the level set matrix for the under part. Third, the DGF was proposed to detect the tongue edge and segment the tongue region in the image, such that the geodesic flow was evaluated in the under part, and the Geo-GVF was evaluated in the upper part.

### Tongue region detection

Before the initialization of the active contour, the tongue region in the clinical image was detected. We used the saliency object detector proposed by Feng et al. [23]. This detector used the entire image as the context, which agreed with human intuition. The tongue body was the deep red region in the tongue image, which corresponded to the brightest region in the saliency map (Figure 2, bottom row). The saliency window composition cost function was defined in Feng et al. [23]. In particular, the algorithm detected two windows with the highest saliencies, and the one with the smaller size was adopted for the tongue body segmentation.

### Initialization

#### Contour initialization

**Figure 3:**
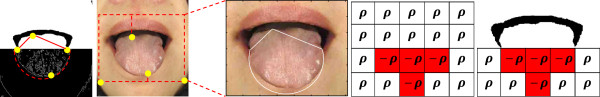
**Tongue image initialization.** From left to right: first, the four special tongue points corresponding to the first image in Figure 2; second, rescaling of the refined tongue image depending on the special tongue points; third, contour initialization; fourth, the level set function (matrix) is initialized for the under part of the tongue image with a negative constant (*ρ* > 0) inside the contour (red region) and a positive value outside the contour; fifth, image denoting the region initialization with the binary image for the upper part.

Based on the refined tongue image, the active contour was initialized depending on the extracted feature points on the image [19]. As illustrated in Figure 3, the under half contour of the first image was constructed based on three special points: two angular points and the tongue tip. The upper half contour was replaced by straight lines, and another point (tongue root) was added to guarantee that the contour was inside the tongue body. The two angular points were automatically detected by the Harris detector while the tongue tip and root were located by edge detectors. The geodesic curve would shrink to the real boundary when the selected tip point was below the real tongue tip. Once the direction was guaranteed, the curve would not stop until the real boundary was reached.

For some tongue images, part of the patient’s nose might be included in the refined window (Figure 2, third image), thereby influencing the propagation of the proposed Geo-GVF. To solve this problem, we obtained the area of the dark region between the tongue root and the upper lip in the binary image (Figure 3), and assumed that the thickness of the upper lip (its height in the image) was at most twice that of the dark region. We then cut off the additional region above the upper lip. Although this refinement may not be accurate, we focused on the extraction of the tongue body rather than other parts.

Overall, owing to the robust ability of the DGF, only the rough locations of these four points were required to confirm that the direction of the initial curve was maintained during propagation. For the upper half contour, it swelled, while for the under part, it shrank. Any inaccuracy caused by the window detection or contour initialization could be overcome as long as the propagating direction was guaranteed.

#### Region initialization

We divided the whole region into two parts according to the row of angular points (Figure 3, fifth image). In the upper part, the curve over-learned or became stuck at some local minimum points, and we adopted the binary image and solved the problem completely. For the under part, we initialized the level set function with a negative constant inside the contour, but a positive value outside the contour, to keep the geodesic curve shrinking to the real boundary.

### Double geo-vector flow (DGF)

#### Geodesic flow

The geodesic model evolves the curve through the propagation of the level set function, which was introduced by Osher and Sethian [24]. The initial contour is represented by a zero level set function: *C*(*t*) = {(*i,j*) | (Φ)(*t,i, j*) = 0}. The curve propagation equation is:

(1) $$\frac{\partial^{\Phi }}{\partial t}+F\left|\nabla \right)\Phi \left|=0,\right)$$

where *F* is the speed function, which depends on the image data and the level set function (Φ). In the geodesic model, the level set function is approximated to the signed distance function (matrix) with |∇*Φ*| = 1. Geodesic flow is introduced as a speed function *F*_*t*_ = (*c* + *κ*), where *κ* is the curvature $\kappa =\mathrm{div}\left(\frac{\nabla \Phi }{\left|\nabla \Phi \right|}\right)$ and *c* is a positive constant to keep the geodesic flow positive. The geodesic model can be rewritten as:

(2) $$\begin{array}{l} \frac{\partial \Phi }{\partial t}=\left|\nabla \Phi \right|\mathrm{div}(g\left(I\right)\frac{\nabla \Phi }{\left|\nabla \Phi \right|})=g(I)\left|\nabla \Phi \right|\kappa +\nabla g(I)\nabla \Phi ,\\ \;g=\frac{1}{1+{\left|\nabla G_{\sigma }*I\right|}^{2}} \end{array}$$

where *I* is an image, and *g*(*I*) is the edge detector. *G*_*σ*_is a two-dimensional Gaussian function with standard deviation *σ*, and is used to smooth the image noise. When the curve is close to the real boundary, *g*(*I*) is approaching zero.

In the traditional level set method, the signed distance function must be re-initialized every several iterations.

The Gateaux derivative [25] is adopted with the functional $\epsilon :\frac{\partial \Phi }{\partial t}=-\frac{\partial \epsilon }{\partial \Phi }$ to simplify this mode. It transfers the energy minimization to the minimization of the functional *ϵ. ϵ* is defined as its variational formulation: ϵ(Φ) = *μP*(Φ) + *ϵ*_*m*_(Φ). *P*(Φ) is a metric to characterize how close a function is to a signed distance function: $P\left(\Phi \right)=\int_{\Omega }\frac{1}{2}{\left(\left|\nabla \Phi \right|-1\right)}^{2}\mathrm{dxdy}$. By calculating the Gateaux derivative of the functional *ϵ* in (1), the final geodesic model can be obtained [26]:

(3) $$\begin{array}{l} \frac{\partial^{\Phi }}{\partial t}\mu \left[\Delta \Phi -\mathrm{div}\left(\frac{\nabla \Phi }{\left|\nabla \Phi \right|}\right)\right]+\lambda \delta \left(\Phi \right)\mathrm{div}\left(g\left(I\right)\frac{\nabla \Phi }{\left|\nabla \Phi \right|}\right)\\ \;+\mathrm{vg\delta}\left(\Phi \right), \end{array}$$

where Δ is the Laplacian operator.

Since the direction of the geodesic flow is fixed, we need the under half curve to shrink. The signed distance function needs to be initialized as a *row × col* matrix (where *row* and *col* denote the image row and column, respectively), with a negative constant (*ρ* > 0) inside the contour (red region) and a positive value outside the contour (Figure 3):

(4) $$\Phi_{\mathrm{un}}=\left\{\begin{array}{ccc} -\rho ,\left(i,j\right) & \in {\text{Ω}}_{\mathrm{un}} & -\partial {\text{Ω}}_{\mathrm{un}}\\ 0, & \left(i,j\right) & \in \partial {\text{Ω}}_{\mathrm{un}}\\ \rho , & \text{Ω} & -{\text{Ω}}_{\mathrm{un}} \end{array}\right),$$

Supposing Ω_*un*_ is a subset in the image domain Ω (Figure 3), then ∂Ω_*un*_ denotes all the points on the boundaries of Ω_*un*_. *ρ* > 0 is a constant for initializing the function ΦΩ_*un*_. In this way, the direction of Ω_*un*_ points to the negative side, or the inside of the contour. Thereby, the curve will shrink during propagation:

(5) $$\begin{array}{ll} \frac{\partial {\text{Φ}}_{\mathrm{un}}}{\partial t}= & \mu \left[\Delta {\text{Φ}}_{\mathrm{un}}-\mathrm{div}\left(\frac{\nabla {\text{Φ}}_{\mathrm{un}}}{\left|\nabla {\text{Φ}}_{\mathrm{un}}\right|}\right)\right]\\ & +\lambda \delta \left({\text{Φ}}_{\mathrm{un}}\right)\mathrm{div}\left(g\left(I\right)\frac{\nabla {\text{Φ}}_{\mathrm{un}}}{\left|\nabla {\text{Φ}}_{\mathrm{un}}\right|}\right)\\ & +\mathrm{vg\delta}\left({\text{Φ}}_{\mathrm{un}}\right). \end{array}$$

**Figure 4:**
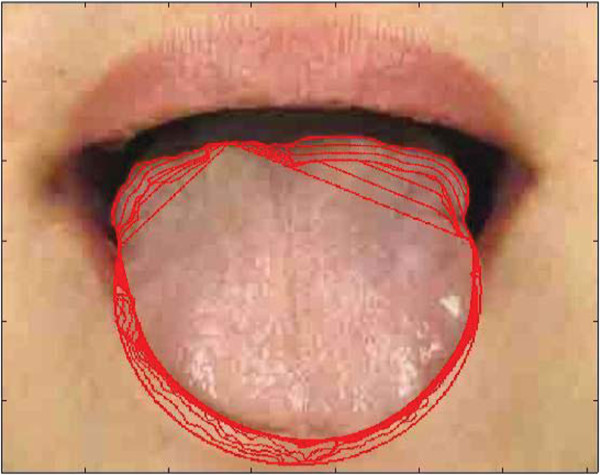
**Intermediate propagating result corresponding to Figure **3**.**

For numerical implementation, since the propagating result is used for evaluating the GVF, we only need the under part to shrink. *Φ*_*un*_^*k*^ is the *k*th iterated level set matrix, with its element being *ϕ*_*i*,*j*_, and then its *k* + 1th iterated form is:

(6) $$\phi_{i,j}^{k+1}=\left\{\begin{array}{c} \phi_{i,j}^{0}\\ \phi_{i,j}^{k}+\mathrm{\tau S}\left(\phi_{i,j}^{k}\right), \end{array}\right)\begin{array}{c} 0<j<\mathrm{sline}\\ \mathrm{sline}<j<\mathrm{row} \end{array},$$

where *S*(*ϕ*_*i*,*j*_^*k*^) corresponds to the right hand side in (5) with *τ* being the stepsize controller. *sline* denotes the partition line between the two parts, and is the row number of the angular point. The intermediate propagating result is shown in Figure 4.

#### Geo-gradient vector flow (Geo-GVF)

The traditional parametric ACM is sensitive to the contour initialization, and the real boundary can only be reached when the initial contour is close to it [14]. In a different manner, the GVF obtains a continuous gradient space by solving a set of vector equations of thermal expansion [27]. Local gradient vectors are not only determined by themselves, but also by their neighboring gradients, and the GVF can overcome the recesses and obstacles during its evolution and converge to the real boundary from a far distance. It is defined as follows:

(7) $$E\left(V_{I}\right)=\omega \left(\left|\nabla I\right|\right)\nabla^{2}V_{I}-h\left(\left|\nabla B\right|\right)\left(V_{1}-\nabla I\right),$$

where *V*_*I*_ is a two-dimensional vector of the GVF, and (*i,j*) *V*_*I*_ (*p*) = (*u*_*I*_ (*p*), *v*_*I*_ (*p*)), *p* = (*i,j*). *I* is an image, and ∇*I* points to the image edge. ^*ω*^ (|∇*I*|) ∇^2^*V*_*I*_ is the smoothing term. According to this objective function, when ∇*I* is close to zero, the area is nearly a constant, and *E*(*V*_*I*_) is mainly dominated by the partial derivatives of the vector field. When the curve is close to the real edge, ∇*I* becomes large. The second item dominates the evolution and drives the curve to the real boundary until *V*_*I*_ – ∇*I*.

Despite the superior performance of the GVF, over-learning can easily occur during the curve evolution [15-17]. To solve this problem, we replace the gray image *I* with the binary image *B* (Figure 3) in (7):

(8) $$E\left(V_{B}\right)=\omega \left(\left|\nabla B\right|\right)\nabla^{2}V_{B}-h\left(\left|\nabla B\right|\right)\left(V_{B}-\nabla B\right).$$

The gradient vectors are a constant value on either the side of the dark region or the tongue root, and the tractive forces toward the two sides are the same, which prevents the curve from over-learning and ensures its accurate convergence. The weighting function *ω* ( • ) is chosen as a constant *ω* (∇*B*) = *w*, indicating that the whole image is being smoothed. *h* ( • ) is chosen as *h* (|∇*B*|) = |∇*B*|^2^, which is related to the strength of the edge.

Based on the above, the active contour in the binary GVF model is calculated according to the following equation:

(9) $$\frac{\partial V_{B}\left(p\left(x,y\right),t\right)}{\partial t}=w\nabla^{2}V_{B}-{\left|\nabla B\right|}^{2}\left(V_{B}-\nabla B\right).$$

It evolves in the upper part, where *y* < *sline*, *y* is the number of rows in the image, and *sline* is the separated line between the two parts.

Referring to (6), the geodesic flow only worked for the under part, and the curve in the upper part held on. In the proposed binary GVF, there was no force in the under part. The geodesic flow ensured the convergence of the under curve, whereas the gradient flow ensured the convergence of the upper curve. In real implementation, after the geodesic propagation, we obtained the final level set function, which provided the contour information inside. To unpack it, we calculated the one-dimensional Dirac measure *δ*_0_ of (6). The active contour is denoted by *C*(*p*), where *p* = *p*(*i*, *j*) denotes the image coordinates:

(10) $$C\left(p\right)=\delta_{0}\left({\text{Φ}}_{\mathrm{un}}\left(p\right)\right)=\frac{d}{\mathrm{dp}}{\text{Φ}}_{\mathrm{un}}\left(p\right),p=p\left(i,j\right).$$

Taking *C* (*i, j*) as the initial active contour of the GVF, we obtain the propagating equation [27]:

(11) $$C\left(p,t\right)=\alpha C^{''}\left(p,t\right)-\beta C^{''''}\left(p,t\right)+V_{B}.$$

In real implementation, considering that the initial contour in the upper part is too far to propagate to the real boundary, a geometric term $G_{B}=g_{B}\times \vec{n}$ is introduced to (11) to help the initial curve quickly converge to the neighborhood of the real boundary, like (2): $g_{B}=\frac{1}{1+{\left|\nabla G_{\sigma }*B\right|}^{2}}$, while $\vec{n}$ denotes the outward normal direction of the curve:

(12) $$C\left(p,t\right)=\alpha C^{''}\left(p,t\right)-\beta C^{''''}\left(p,t\right)+\max\left(V_{B},G_{B}\right).$$

When the curve is far away from the real boundary, *V*_*B*_ is nearly zero, while *g*_*B*_ is close to 1, and the curve is propagating as illustrated in Figure 4. When the curve is close to the tongue edge, *V*_*B*_ will dominate the propagation and adjust the curve to the real boundary of the tongue body. This new scheme is called the Geo-GVF.

**Figure 5:**
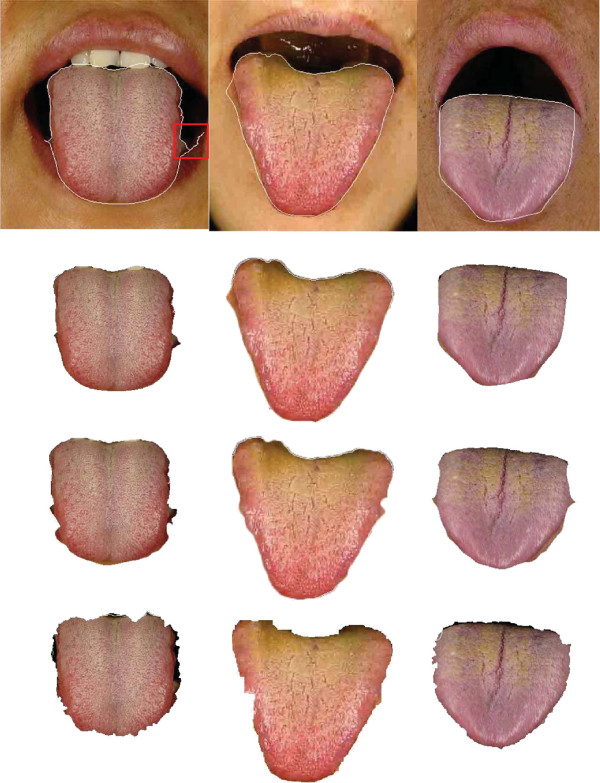
**Examples of tongue image segmentation.** Top row: refined tongue images and final propagating results of the proposed DGF (white curves); second row: corresponding segmentation results of the first row; third row: segmentation results of C^2^G^2^FSnake [19]; fourth row: segmentation results of double snakes [17].

The DGF was implemented in MATLAB, and the source codes can be downloaded from Additional file 1. We initialized the tongue image by adopting the saliency object detection. The region of the tongue body was refined by a rectangular window (Figure 2, top row). The contour initialization used the special shape of the tongue body and its location on the face. The parameter settings were *μ* = 1, *λ* = 3, and *ν* = 0*.*5 for the geodesic flow. *μ* was the coefficient of the internal (penalizing) energy term $\text{Δ}{\text{Φ}}_{\mathrm{un}}-\mathrm{div}\left(\frac{\nabla \Phi_{\mathrm{un}}}{\left|\nabla \Phi_{\mathrm{un}}\right|}\right)$, while *λ* represented the coefficient of the weighted length term $\delta {\text{Φ}}_{\mathrm{un}}\mathrm{div}\left(g\left(I\right)\frac{\nabla \Phi_{\mathrm{un}}}{\left|\nabla \Phi_{\mathrm{un}}\right|}\right)$. *ν* denoted the coefficient of the weighted area term *gδ*(*Φ*_*un*_), and generally, a small *ν* was chosen. For the Geo-GVF, its viscosity parameters were *α* = 1, *β* = 1. Some examples of the tongue segmentations are shown in Figure 5.

We compared the DGF with our previous work C^2^G^2^FSnake [19], Zhai et al. [17], and Fu et al. [15]. Zhai et al. [17] adopted the H-channel as an initial contour for the outside Snake contour, and a double snake was carried out afterwards. The inside contour kept propagating as long as it did not meet the outside contour. The outside contour kept the inside curve close to the real boundary without over-learning as a barrier. Fu et al. [15] introduced AdaBoost to locate the tongue body in the human face, and then eliminated the H-channel, which was regarded as the same as the lip and skin color. The initial contour was obtained close to the real boundary through a polar edge detector, and the Snake was evolved afterwards.

### Dataset evaluation and error measurements

The clinical tongue image database was provided by Shanghai University of TCM, and categorized by tongue color as follows: light white tongue (tinge, light white); red tongue (red, deep red, deep purple); purple tongue (heliotrope, pompadour, heliotrope, purple, modena); and carmoisine tongue. There were four classes and ten branches. Ten images were chosen for each branch, giving 100 images in total. Some examples are shown in Figure 2. The entire dataset can be downloaded from Additional file 2.

This study has been approved by the Shanghai society of medical ethics. All the patients have signed the informed consent form.

Boundary error metrics and area error metrics were used to evaluate the performance of the tongue image segmentation. For the boundary error metrics, the Hausdorff distance (HD) and the mean distance (MD) to the closest point were adopted to measure the errors between the proposed automatic segmentation result and the manual contour [19]. We used the normalized terms of the two distances (norm.HD and norm.MD) as the boundary error metrics. For the area error metrics, referring to Shi et al. [19], the false-positive (FP) volume fraction, false-negative (FN) volume fraction, and true-positive (TP) volume fraction were adopted.

## Results and discussion

In this paper, a computerized tongue image segmentation approach, the DGF, was proposed for tongue diagnosis. We adopted a saliency object detector to refine the clinical tongue image. By taking advantage of the prior knowledge of the tongue image, the DGF was evaluated in the under and upper parts of the image, respectively. The two curves in the DGF precisely extracted the tongue region.

### Color map and geometric term

**Figure 6:**
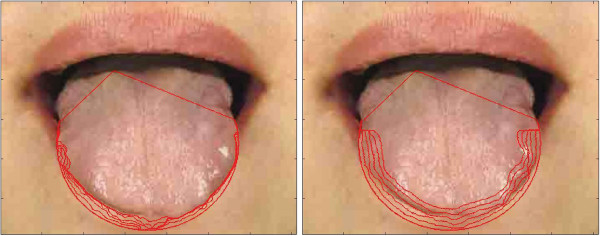
Geodesic propagating results for the gray map and H-channel map.

**Figure 7:**
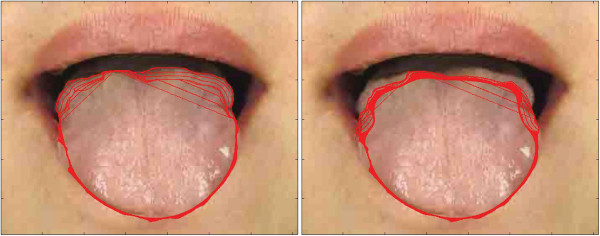
Geo-GVF propagating results for the binary map and gray map.

The tongue color map has been highly analyzed for various types of information in tongue diagnosis [28]. In this section, we analyzed the color map in the tongue segmentation. The H-channel, gray map, and binary map were used for different active contour propagations. Comparisons are shown in Figures 6 and 7. We also provided the propagating results with or without the geometric term, to show the role of the geometric term in the propagation of the Geo-GVF.

#### H-channel map

In our study, we found that the H-channel map was not always beneficial for tongue image segmentation. Figure 6 shows the geodesic propagating results for both the gray map (left image) and H-channel map (right image). If the face color in its H-channel was close to the tongue color, the edge detector $g_{H}=\frac{1}{1+|\nabla \mathrm{G\sigma}*H|{}^{2}}$ (*H* denotes the H-channel map) could not distinguish the tongue body from the image.

#### Binary map

In this study, we initialized the upper part as a binary image (Figure 3). In this manner, *∇B* (8) was a constant at both sides of the tongue root, the tractive forces from the two sides were the same, and no over-learning or local minima occurred. We compared the Geo-GVF propagating results in both the gray map and the binary map (Figure 7). It can be seen that, if the binary map was not used, local minima would occur (right image).

#### Geometric term

**Figure 8:**
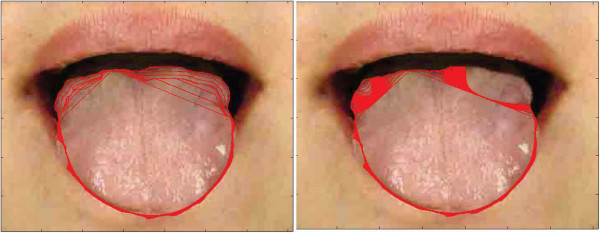
GVF propagating results with or without the geometric term.

Referring to (12), when the curve was far away from the real boundary, *V*_*B*_ was nearly zero, and we adopted the geometric term *G*_*B*_ to propagate the curve along its outward direction. Meanwhile, when the curve was close to the tongue edge, *V*_*B*_ dominated the propagation and adjusted the curve to the real boundary of the tongue body. If we did not adopt the geometric term during the propagation of the GVF (Figure 8, right image), the initial contour in the upper part was too far, and no external force *V*_*B*_ in (11) could be found around the initial contour.

### Overall comparison

In this section, the DGF was compared within the dataset with different classes. The overall performances were presented afterwards in comparison with other representative studies [15,17,19].

#### Class variation

We have summarized the detailed segmentation results with respect to each tongue class in Table 1. The results showed that the performance of the DGF was inferior for the red tongue class compared with the other classes. This arose because the tongue color was close to the lip color in the red tongue class, making it hard for the DGF to converge to the real boundary, and leading to possible over-learning during the curve propagation. However, in the light white and carmoisine tongue classes, the tongue color was clearly distinct from the lip color, and a deep valley in the level set space was formed between them to prevent the curve propagating further, thereby providing superior performances in these two classes. Despite the slight differences in these results, the performances were good in the four classes, suggesting the scalability and effectiveness of the proposed DGF.

**Table 1:** Detailed segmentation results with respect to each tongue class

**Tongue color**	**Norm. HD (%)**	**Norm. MD (%)**	**FN (%)**	**FP (%)**	**TP (%)**
Light white	0.07	1.17	1.4	1.15	98.95
Red	0.57	1.62	1.47	2.17	97.82
Purple	0.36	1.91	1.17	1.92	98.08
Carmoisine	0.16	1.02	1.64	0.80	99.21

#### Comparative experiments

**Figure 9:**
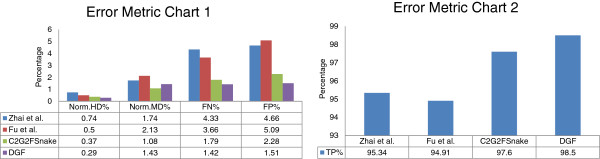
**Tongue image segmentation results.** The smaller Norm.HD%, Norm.MD%, FN%, and FP% are, the better they will be. The larger TP% is, the better it will be.

Comparative tests using the tongue image database in this study are shown in Figures 5 and 9. In Figure 5, we show visual comparisons of the DGF and two previous studies [17,19]. Specifically, the top row denotes the refined tongue images and the final propagating curves of the DGF (white curves), the second row corresponds to the segmentation results of the first row, the third row denotes the segmentation results of C2G2FSnake [19], and the fourth row denotes the segmentation results using double snakes [17]. Compared with Zhai et al. [17] and Shi et al. [19], the tongue edge extracted by the DGF was smoother and more accurate.

The DGF exhibited a better performance on almost every metric except for Norm.MD%, which measured the mean distance between the edges obtained automatically and the standard tongue body boundaries obtained manually (Figure 9), and was affected when the point-to-point distance was calculated. The performance for Norm.MD% was weaker because, during the propagation of the geodesic active contour, some local minimum points needed to be overcome. Consequently, the curve split and merged again, which left unnecessary tails in the final result (Figure 5, marked by the red frame in the first image).

In C^2^G^2^FSnake [19], color space information was introduced to control both the geometrical model and the parameterized GVF model to realize a seamless switch from rough evolution to refined evolution. However, the parameters for different tongue color categories had to vary to adapt to the color contrast between the tongue body and the facial skin. Besides, for some special images, the active contours were initialized incorrectly for the evolution of C^2^G^2^FSnake. In this study, the geodesic model could propagate to the real boundary by splitting itself.

#### Computational complexity

The DGF was evaluated in the MATLAB environment (MATLAB 7.9) on the Windows 7 system, with Intel Core 2 CPU and 2 GB of RAM. The curve movement in the geodesic model was realized by updating of the level set matrix, Φ_*m×n*_, where *m*and *n*denoted the width and height of the matrix, or the width and height of the image. The level set matrix was updated at every iteration, and thus the computation complexity for the geodesic flow was *O*(*mn*). On the other hand, the curve movement of the GVF was updated by every point on the curve at every iteration. Suppose that the length of the curve was *l*, the evolution of the GVF shall be performed in time *O*(*l*). Usually, *l* < < *mn*. The DGF was a combination of the geodesic flow and the GVF, and it would cost a bit more time than the GVF (or Snake) model used in previous studies [15,17,19].

## Conclusion

In this study, the DGF showed efficiency and effectiveness for automatic tongue image segmentation.

## Abbreviations

TCM: Traditional Chinese medicine; DGF: Double geo-vector flow; ACM: Active contour model; Geo-GVF: Geo-gradient vector flow; GVF: Gradient vector flow; HD: Hausdorff distance; MD: Mean distance; FP: False-positive volume fraction; FN: False-negative volume fraction; TP: True-positive volume fraction.

## Competing interests

The authors declare that they have no competing interests.

## Authors’ contributions

MJS and GZL conceived and designed the study. MJS and FFL performed the experiments. MJS, GZL, and CX revised the paper. All authors read and approved the final manuscript.

## Supplementary Material

Additional file 1 **Source codes of the DGF in MATLAB language.** Please refer to readme in the zip files. Click here for file

Additional file 2 **The collection dataset of tongue images and segmentation benchmarks.** Please refer to the subsection entitled Dataset evaluation and error measurements. Click here for file
