# Supplementary figures and images for: Computerized tongue image segmentation via the double geo-vector flow
Source: Chin Med. 2014 Feb 8;9:7. doi: 10.1186/1749-8546-9-7 (PMC3922256; doi:10.1186/1749-8546-9-7)

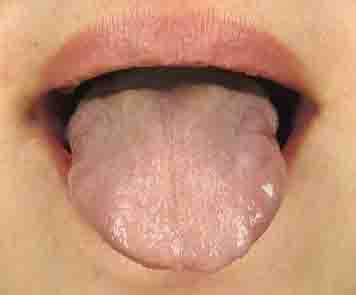

Supplement: Additional file 1 — Source codes of the DGF in MATLAB language. Please refer to readme in the zip files. [file 1749-8546-9-7-S1.zip › DGF/DGF/test.jpg]

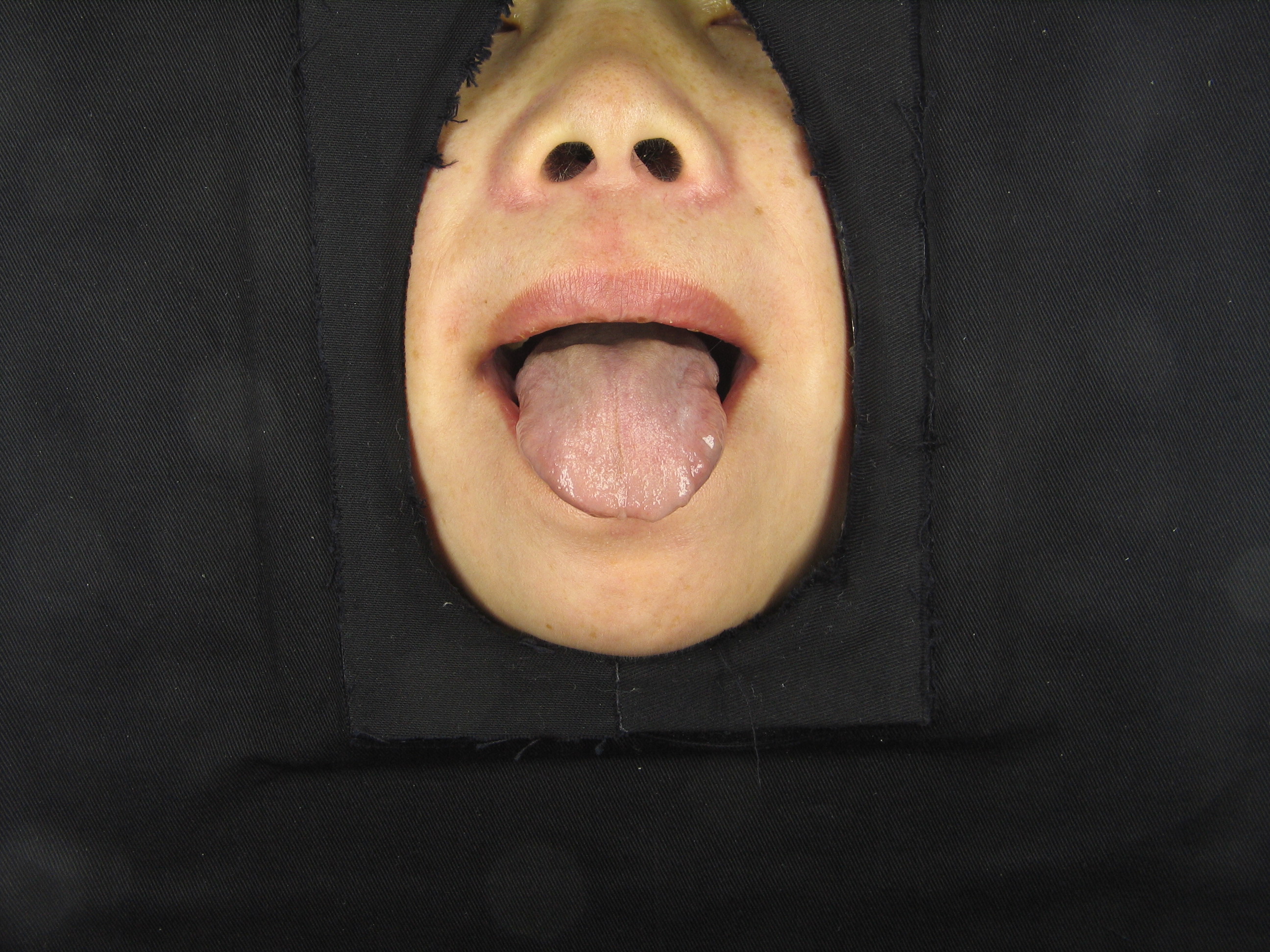

Supplement: Additional file 1 — Source codes of the DGF in MATLAB language. Please refer to readme in the zip files. [file 1749-8546-9-7-S1.zip › DGF/Salience Detection/T003.JPG]

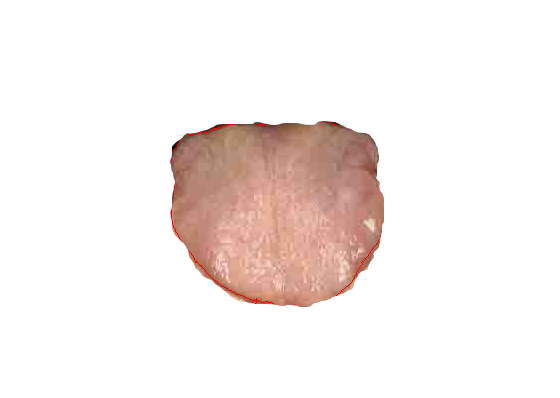

Supplement: Additional file 2 — The collection dataset of tongue images and segmentation benchmarks. Please refer to the subsection entitled Dataset evaluation and error measurements. [file 1749-8546-9-7-S2.zip › Stanard images/1.tif]

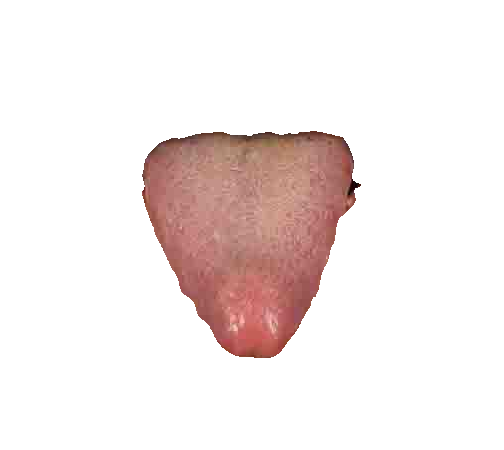

Supplement: Additional file 2 — The collection dataset of tongue images and segmentation benchmarks. Please refer to the subsection entitled Dataset evaluation and error measurements. [file 1749-8546-9-7-S2.zip › Stanard images/10.tif]

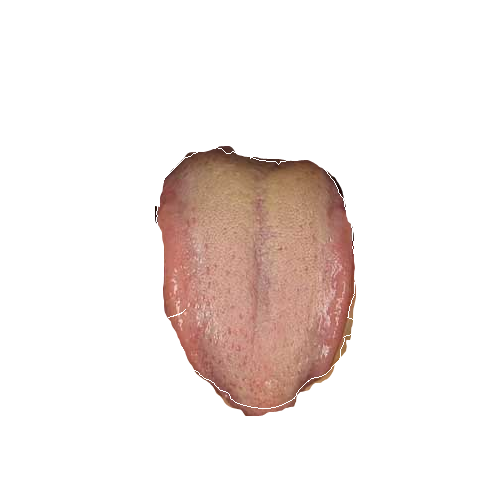

Supplement: Additional file 2 — The collection dataset of tongue images and segmentation benchmarks. Please refer to the subsection entitled Dataset evaluation and error measurements. [file 1749-8546-9-7-S2.zip › Stanard images/12.tif]

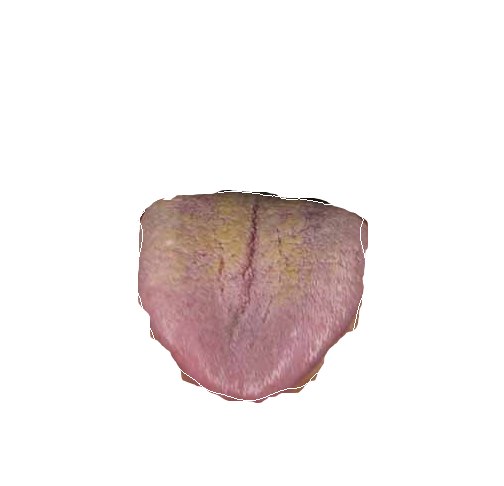

Supplement: Additional file 2 — The collection dataset of tongue images and segmentation benchmarks. Please refer to the subsection entitled Dataset evaluation and error measurements. [file 1749-8546-9-7-S2.zip › Stanard images/13.tif]

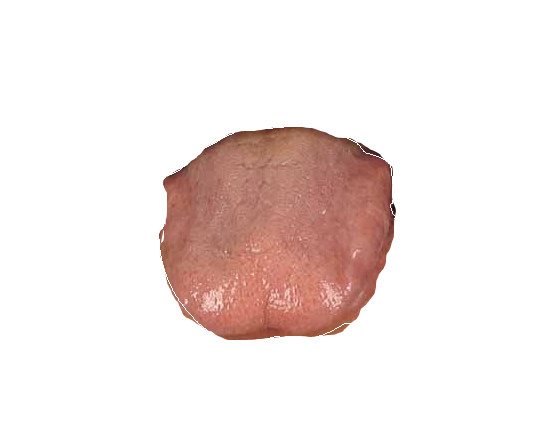

Supplement: Additional file 2 — The collection dataset of tongue images and segmentation benchmarks. Please refer to the subsection entitled Dataset evaluation and error measurements. [file 1749-8546-9-7-S2.zip › Stanard images/14.tif]

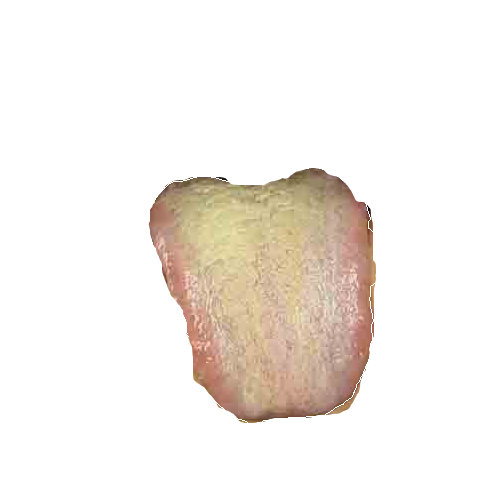

Supplement: Additional file 2 — The collection dataset of tongue images and segmentation benchmarks. Please refer to the subsection entitled Dataset evaluation and error measurements. [file 1749-8546-9-7-S2.zip › Stanard images/15.tif]

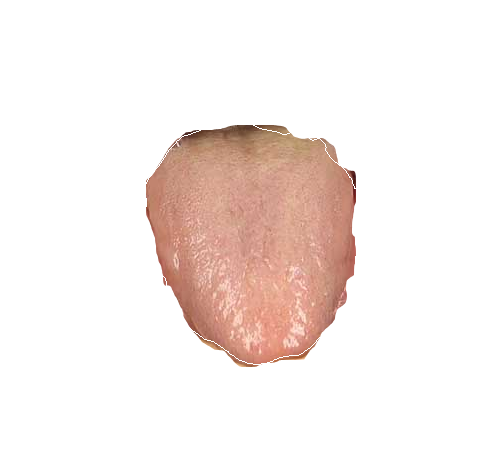

Supplement: Additional file 2 — The collection dataset of tongue images and segmentation benchmarks. Please refer to the subsection entitled Dataset evaluation and error measurements. [file 1749-8546-9-7-S2.zip › Stanard images/16.tif]

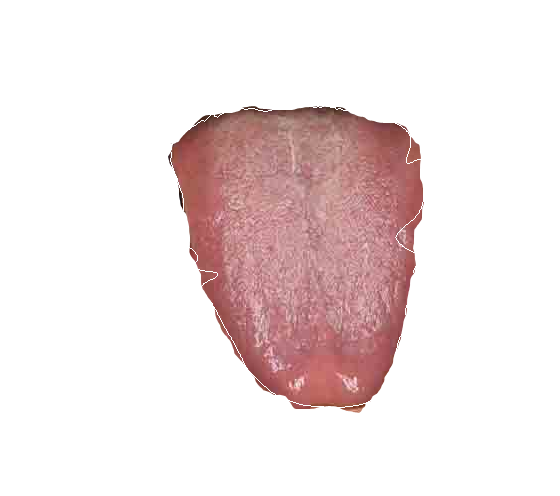

Supplement: Additional file 2 — The collection dataset of tongue images and segmentation benchmarks. Please refer to the subsection entitled Dataset evaluation and error measurements. [file 1749-8546-9-7-S2.zip › Stanard images/17.tif]

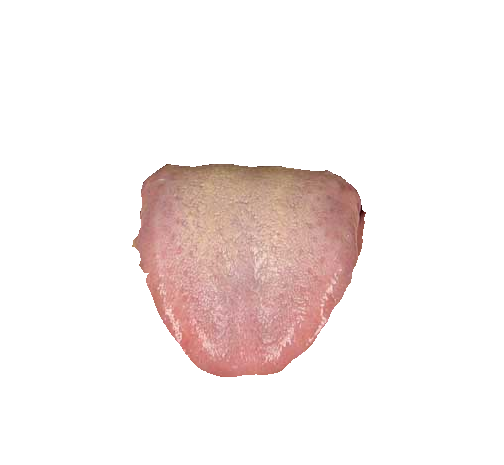

Supplement: Additional file 2 — The collection dataset of tongue images and segmentation benchmarks. Please refer to the subsection entitled Dataset evaluation and error measurements. [file 1749-8546-9-7-S2.zip › Stanard images/19.tif]

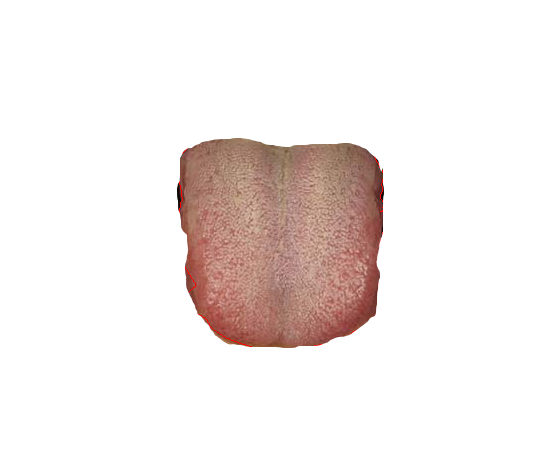

Supplement: Additional file 2 — The collection dataset of tongue images and segmentation benchmarks. Please refer to the subsection entitled Dataset evaluation and error measurements. [file 1749-8546-9-7-S2.zip › Stanard images/2.tif]

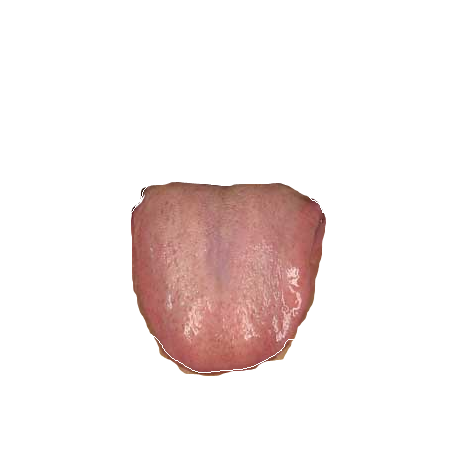

Supplement: Additional file 2 — The collection dataset of tongue images and segmentation benchmarks. Please refer to the subsection entitled Dataset evaluation and error measurements. [file 1749-8546-9-7-S2.zip › Stanard images/20.tif]

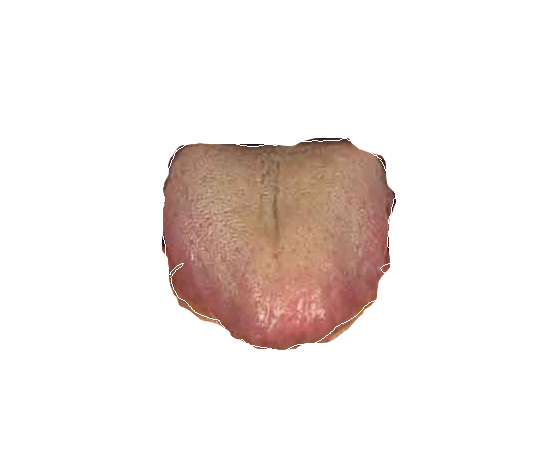

Supplement: Additional file 2 — The collection dataset of tongue images and segmentation benchmarks. Please refer to the subsection entitled Dataset evaluation and error measurements. [file 1749-8546-9-7-S2.zip › Stanard images/21.tif]

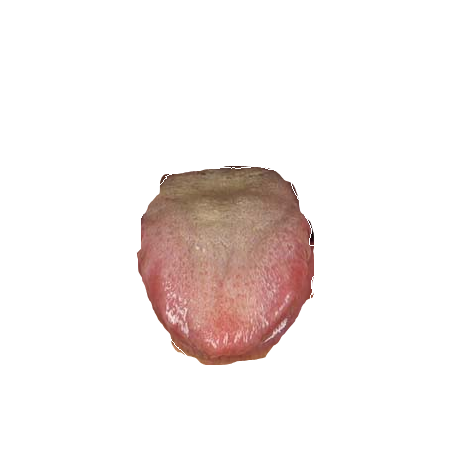

Supplement: Additional file 2 — The collection dataset of tongue images and segmentation benchmarks. Please refer to the subsection entitled Dataset evaluation and error measurements. [file 1749-8546-9-7-S2.zip › Stanard images/22.tif]

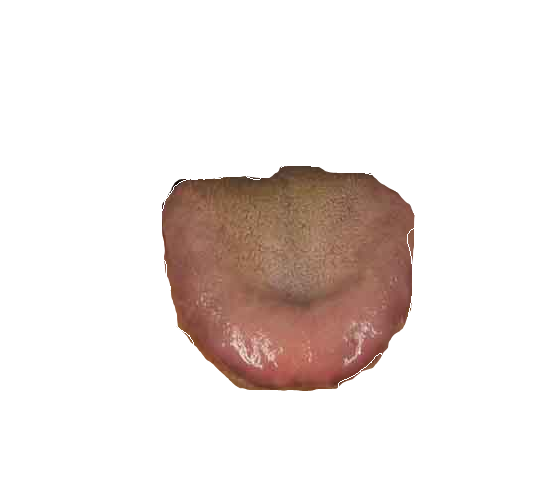

Supplement: Additional file 2 — The collection dataset of tongue images and segmentation benchmarks. Please refer to the subsection entitled Dataset evaluation and error measurements. [file 1749-8546-9-7-S2.zip › Stanard images/23.tif]

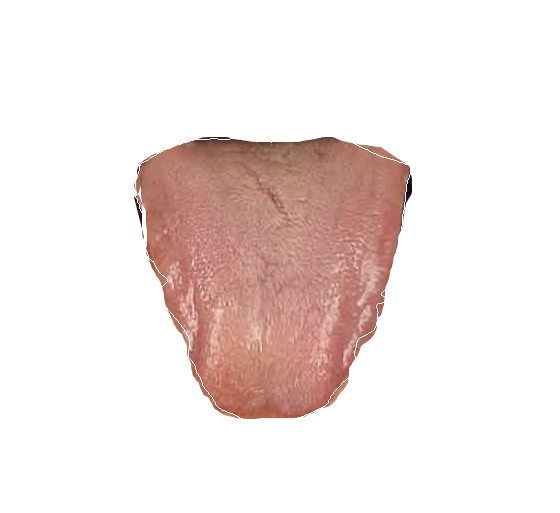

Supplement: Additional file 2 — The collection dataset of tongue images and segmentation benchmarks. Please refer to the subsection entitled Dataset evaluation and error measurements. [file 1749-8546-9-7-S2.zip › Stanard images/24.tif]

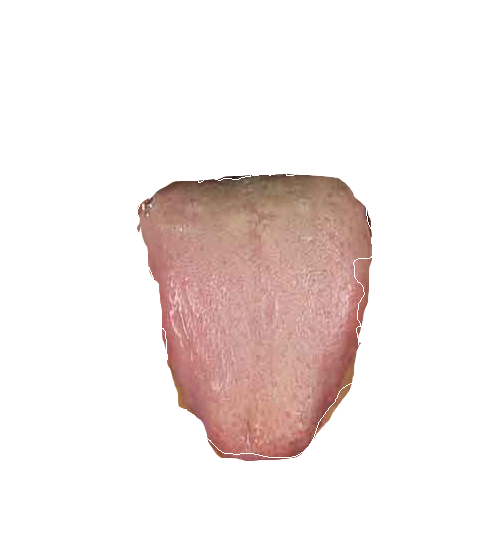

Supplement: Additional file 2 — The collection dataset of tongue images and segmentation benchmarks. Please refer to the subsection entitled Dataset evaluation and error measurements. [file 1749-8546-9-7-S2.zip › Stanard images/25.tif]

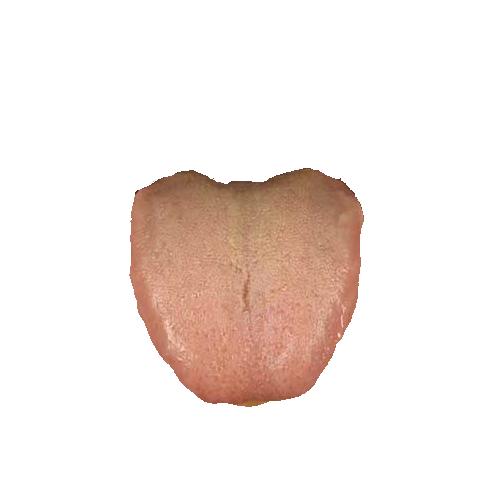

Supplement: Additional file 2 — The collection dataset of tongue images and segmentation benchmarks. Please refer to the subsection entitled Dataset evaluation and error measurements. [file 1749-8546-9-7-S2.zip › Stanard images/27.tif]

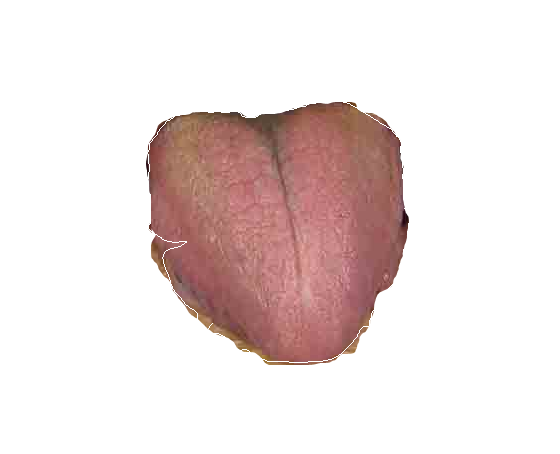

Supplement: Additional file 2 — The collection dataset of tongue images and segmentation benchmarks. Please refer to the subsection entitled Dataset evaluation and error measurements. [file 1749-8546-9-7-S2.zip › Stanard images/28.tif]

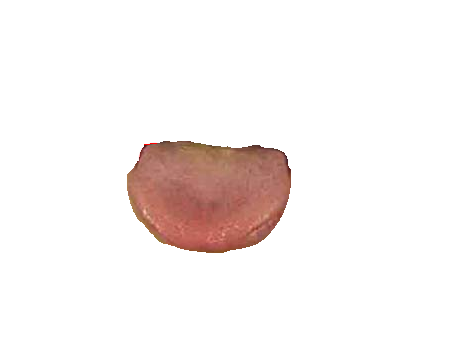

Supplement: Additional file 2 — The collection dataset of tongue images and segmentation benchmarks. Please refer to the subsection entitled Dataset evaluation and error measurements. [file 1749-8546-9-7-S2.zip › Stanard images/29.tif]

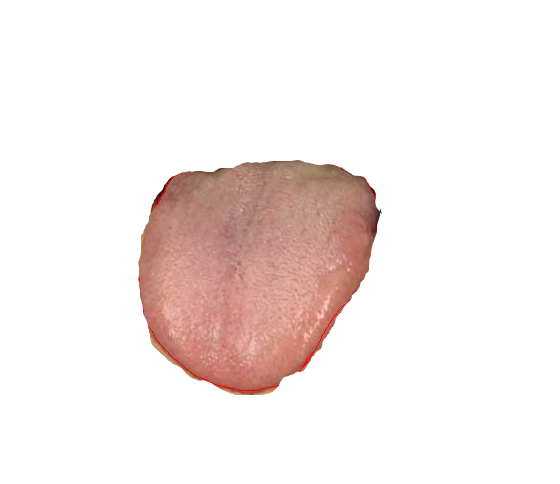

Supplement: Additional file 2 — The collection dataset of tongue images and segmentation benchmarks. Please refer to the subsection entitled Dataset evaluation and error measurements. [file 1749-8546-9-7-S2.zip › Stanard images/3.tif]

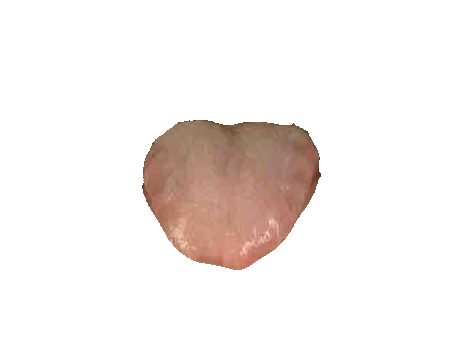

Supplement: Additional file 2 — The collection dataset of tongue images and segmentation benchmarks. Please refer to the subsection entitled Dataset evaluation and error measurements. [file 1749-8546-9-7-S2.zip › Stanard images/30.tif]

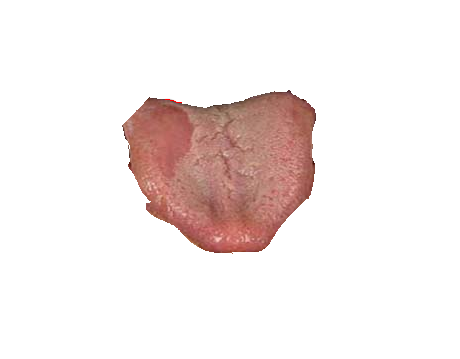

Supplement: Additional file 2 — The collection dataset of tongue images and segmentation benchmarks. Please refer to the subsection entitled Dataset evaluation and error measurements. [file 1749-8546-9-7-S2.zip › Stanard images/31.tif]

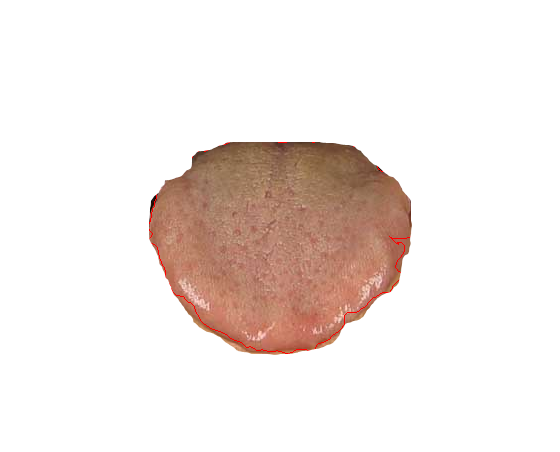

Supplement: Additional file 2 — The collection dataset of tongue images and segmentation benchmarks. Please refer to the subsection entitled Dataset evaluation and error measurements. [file 1749-8546-9-7-S2.zip › Stanard images/4.tif]

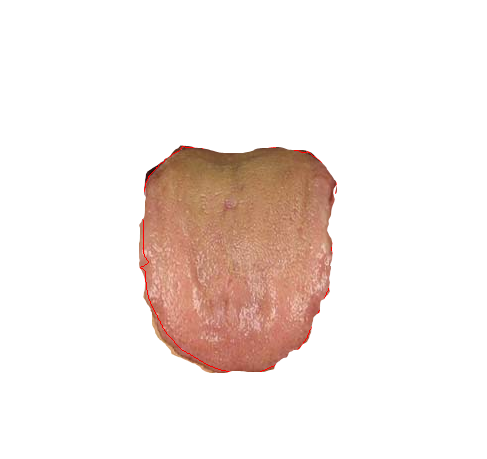

Supplement: Additional file 2 — The collection dataset of tongue images and segmentation benchmarks. Please refer to the subsection entitled Dataset evaluation and error measurements. [file 1749-8546-9-7-S2.zip › Stanard images/5.tif]

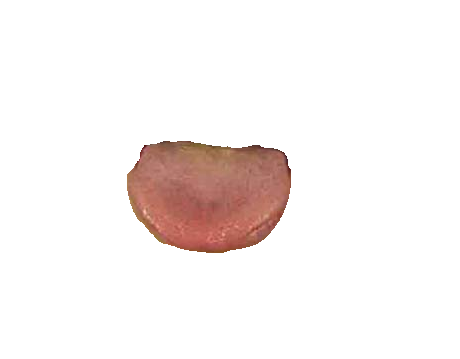

Supplement: Additional file 2 — The collection dataset of tongue images and segmentation benchmarks. Please refer to the subsection entitled Dataset evaluation and error measurements. [file 1749-8546-9-7-S2.zip › Stanard images/6.tif]

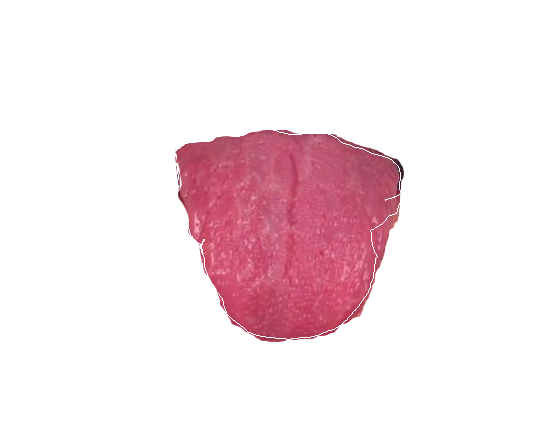

Supplement: Additional file 2 — The collection dataset of tongue images and segmentation benchmarks. Please refer to the subsection entitled Dataset evaluation and error measurements. [file 1749-8546-9-7-S2.zip › Stanard images/7.tif]

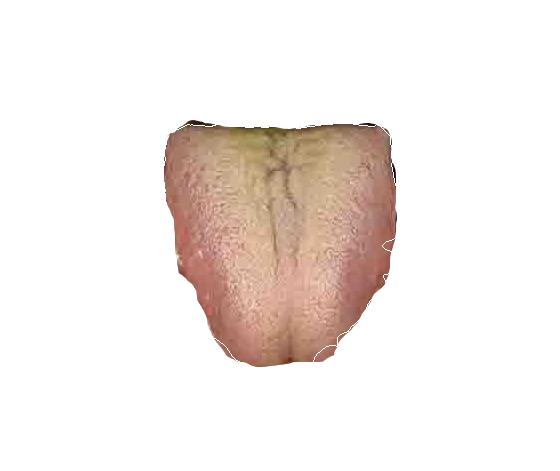

Supplement: Additional file 2 — The collection dataset of tongue images and segmentation benchmarks. Please refer to the subsection entitled Dataset evaluation and error measurements. [file 1749-8546-9-7-S2.zip › Stanard images/8.tif]

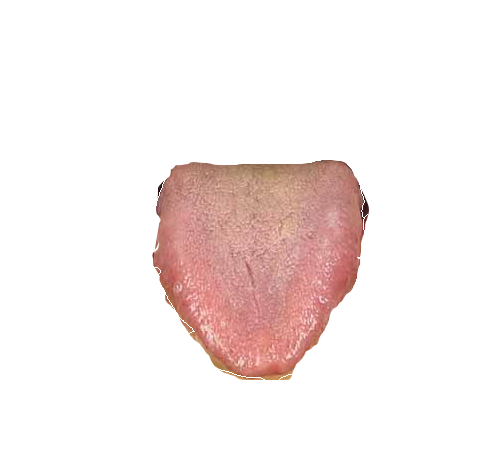

Supplement: Additional file 2 — The collection dataset of tongue images and segmentation benchmarks. Please refer to the subsection entitled Dataset evaluation and error measurements. [file 1749-8546-9-7-S2.zip › Stanard images/9.tif]

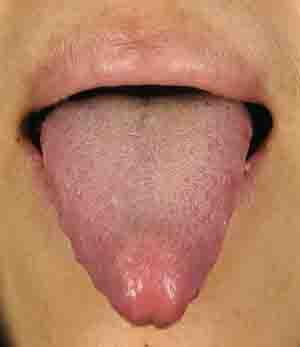

Supplement: Additional file 2 — The collection dataset of tongue images and segmentation benchmarks. Please refer to the subsection entitled Dataset evaluation and error measurements. [file 1749-8546-9-7-S2.zip › Test images/t10.jpg]

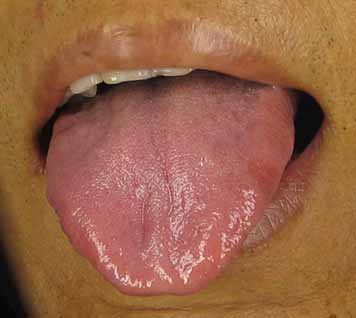

Supplement: Additional file 2 — The collection dataset of tongue images and segmentation benchmarks. Please refer to the subsection entitled Dataset evaluation and error measurements. [file 1749-8546-9-7-S2.zip › Test images/t11.jpg]

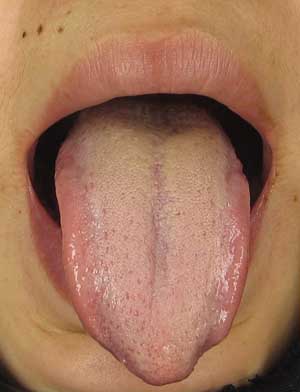

Supplement: Additional file 2 — The collection dataset of tongue images and segmentation benchmarks. Please refer to the subsection entitled Dataset evaluation and error measurements. [file 1749-8546-9-7-S2.zip › Test images/t12.jpg]

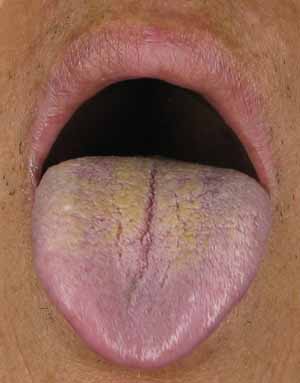

Supplement: Additional file 2 — The collection dataset of tongue images and segmentation benchmarks. Please refer to the subsection entitled Dataset evaluation and error measurements. [file 1749-8546-9-7-S2.zip › Test images/t13.jpg]

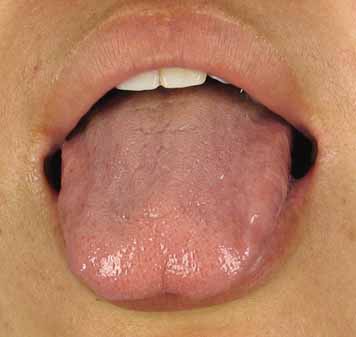

Supplement: Additional file 2 — The collection dataset of tongue images and segmentation benchmarks. Please refer to the subsection entitled Dataset evaluation and error measurements. [file 1749-8546-9-7-S2.zip › Test images/t14.jpg]

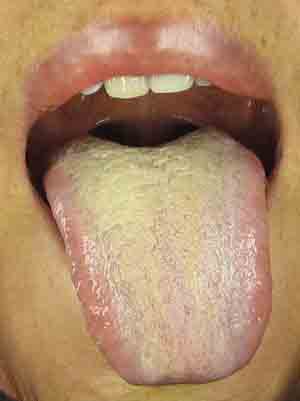

Supplement: Additional file 2 — The collection dataset of tongue images and segmentation benchmarks. Please refer to the subsection entitled Dataset evaluation and error measurements. [file 1749-8546-9-7-S2.zip › Test images/t15.jpg]

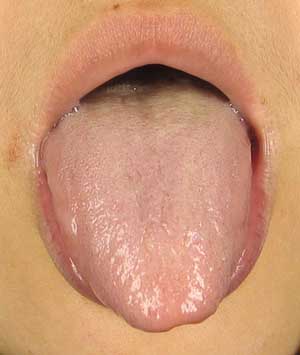

Supplement: Additional file 2 — The collection dataset of tongue images and segmentation benchmarks. Please refer to the subsection entitled Dataset evaluation and error measurements. [file 1749-8546-9-7-S2.zip › Test images/t16.jpg]

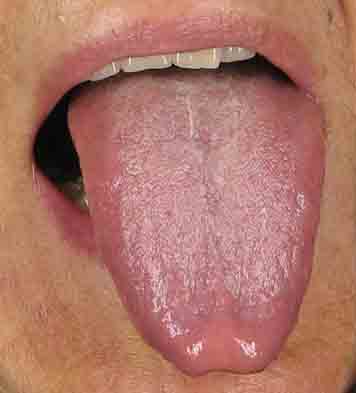

Supplement: Additional file 2 — The collection dataset of tongue images and segmentation benchmarks. Please refer to the subsection entitled Dataset evaluation and error measurements. [file 1749-8546-9-7-S2.zip › Test images/t17.jpg]

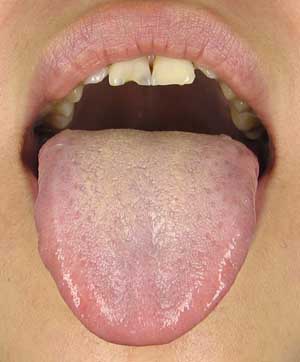

Supplement: Additional file 2 — The collection dataset of tongue images and segmentation benchmarks. Please refer to the subsection entitled Dataset evaluation and error measurements. [file 1749-8546-9-7-S2.zip › Test images/t19.jpg]

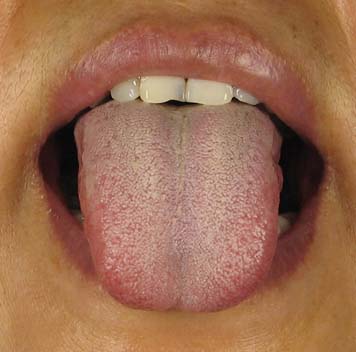

Supplement: Additional file 2 — The collection dataset of tongue images and segmentation benchmarks. Please refer to the subsection entitled Dataset evaluation and error measurements. [file 1749-8546-9-7-S2.zip › Test images/t2.jpg]

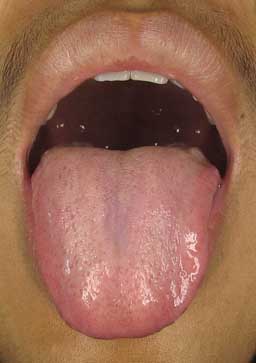

Supplement: Additional file 2 — The collection dataset of tongue images and segmentation benchmarks. Please refer to the subsection entitled Dataset evaluation and error measurements. [file 1749-8546-9-7-S2.zip › Test images/t20.jpg]

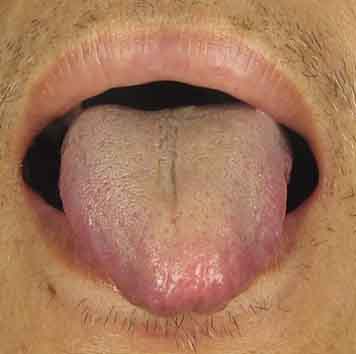

Supplement: Additional file 2 — The collection dataset of tongue images and segmentation benchmarks. Please refer to the subsection entitled Dataset evaluation and error measurements. [file 1749-8546-9-7-S2.zip › Test images/t21.jpg]

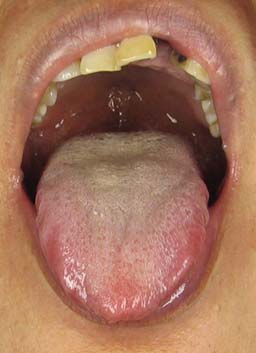

Supplement: Additional file 2 — The collection dataset of tongue images and segmentation benchmarks. Please refer to the subsection entitled Dataset evaluation and error measurements. [file 1749-8546-9-7-S2.zip › Test images/t22.jpg]

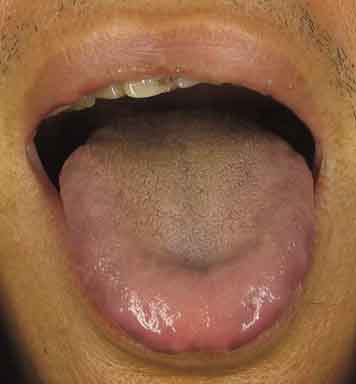

Supplement: Additional file 2 — The collection dataset of tongue images and segmentation benchmarks. Please refer to the subsection entitled Dataset evaluation and error measurements. [file 1749-8546-9-7-S2.zip › Test images/t23.jpg]

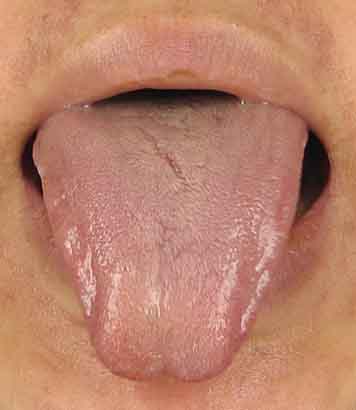

Supplement: Additional file 2 — The collection dataset of tongue images and segmentation benchmarks. Please refer to the subsection entitled Dataset evaluation and error measurements. [file 1749-8546-9-7-S2.zip › Test images/t24.jpg]

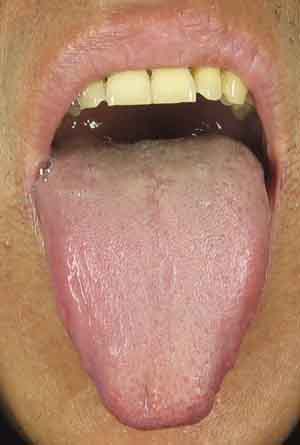

Supplement: Additional file 2 — The collection dataset of tongue images and segmentation benchmarks. Please refer to the subsection entitled Dataset evaluation and error measurements. [file 1749-8546-9-7-S2.zip › Test images/t25.jpg]

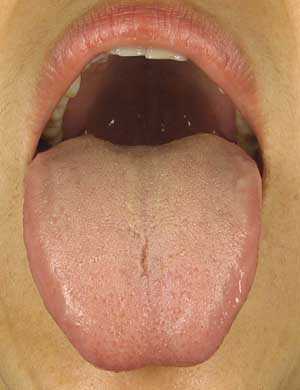

Supplement: Additional file 2 — The collection dataset of tongue images and segmentation benchmarks. Please refer to the subsection entitled Dataset evaluation and error measurements. [file 1749-8546-9-7-S2.zip › Test images/t27.jpg]

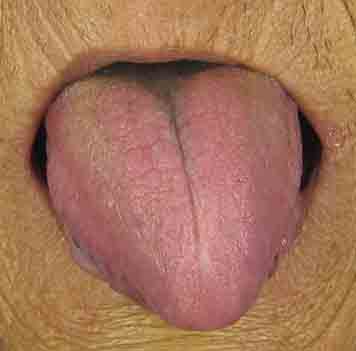

Supplement: Additional file 2 — The collection dataset of tongue images and segmentation benchmarks. Please refer to the subsection entitled Dataset evaluation and error measurements. [file 1749-8546-9-7-S2.zip › Test images/t28.jpg]

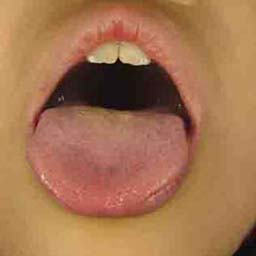

Supplement: Additional file 2 — The collection dataset of tongue images and segmentation benchmarks. Please refer to the subsection entitled Dataset evaluation and error measurements. [file 1749-8546-9-7-S2.zip › Test images/t29.jpg]

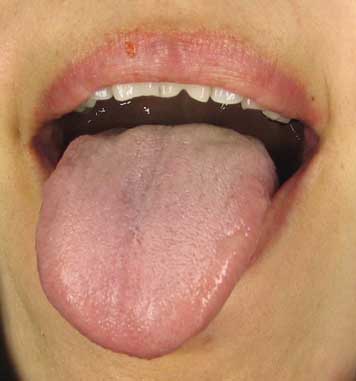

Supplement: Additional file 2 — The collection dataset of tongue images and segmentation benchmarks. Please refer to the subsection entitled Dataset evaluation and error measurements. [file 1749-8546-9-7-S2.zip › Test images/t3.jpg]

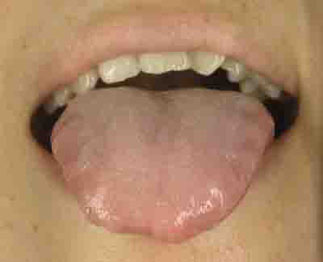

Supplement: Additional file 2 — The collection dataset of tongue images and segmentation benchmarks. Please refer to the subsection entitled Dataset evaluation and error measurements. [file 1749-8546-9-7-S2.zip › Test images/t30.jpg]

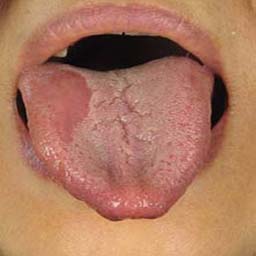

Supplement: Additional file 2 — The collection dataset of tongue images and segmentation benchmarks. Please refer to the subsection entitled Dataset evaluation and error measurements. [file 1749-8546-9-7-S2.zip › Test images/t31.jpg]

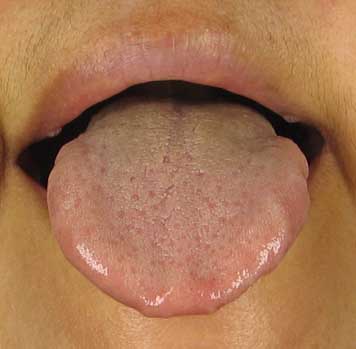

Supplement: Additional file 2 — The collection dataset of tongue images and segmentation benchmarks. Please refer to the subsection entitled Dataset evaluation and error measurements. [file 1749-8546-9-7-S2.zip › Test images/t4.jpg]

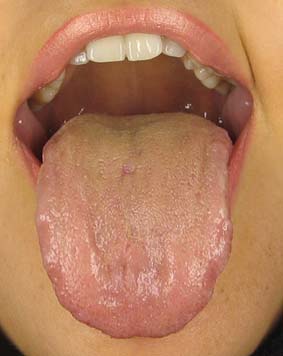

Supplement: Additional file 2 — The collection dataset of tongue images and segmentation benchmarks. Please refer to the subsection entitled Dataset evaluation and error measurements. [file 1749-8546-9-7-S2.zip › Test images/t5.jpg]

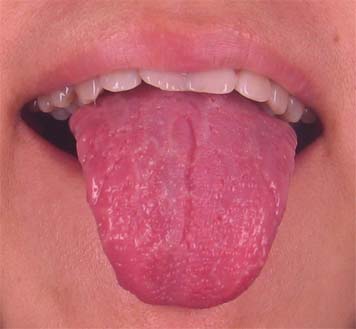

Supplement: Additional file 2 — The collection dataset of tongue images and segmentation benchmarks. Please refer to the subsection entitled Dataset evaluation and error measurements. [file 1749-8546-9-7-S2.zip › Test images/t7.jpg]

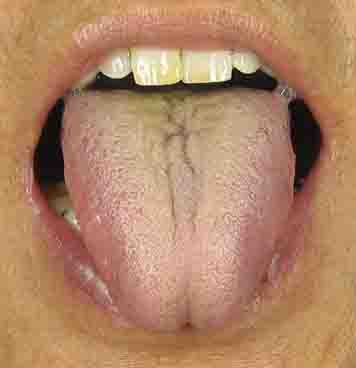

Supplement: Additional file 2 — The collection dataset of tongue images and segmentation benchmarks. Please refer to the subsection entitled Dataset evaluation and error measurements. [file 1749-8546-9-7-S2.zip › Test images/t8.jpg]

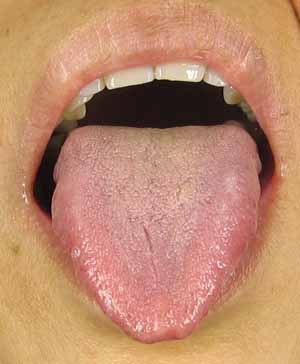

Supplement: Additional file 2 — The collection dataset of tongue images and segmentation benchmarks. Please refer to the subsection entitled Dataset evaluation and error measurements. [file 1749-8546-9-7-S2.zip › Test images/t9.jpg]
